# Z-Scheme Photocatalyst Constructed by Natural Attapulgite and Upconversion Rare Earth Materials for Desulfurization

**DOI:** 10.3389/fchem.2018.00477

**Published:** 2018-10-05

**Authors:** Fengqin Wu, Xiazhang Li, Haiguang Zhang, Shixiang Zuo, Chao Yao

**Affiliations:** Advanced Catalysis and Green Manufacturing Collaborative Innovation Center, Changzhou University, Changzhou, China

**Keywords:** attapulgite, upconversion, rare earth, Z scheme, photocatalyst

## Abstract

The Er^3+^:CeO_2_/ATP (attapulgite) nanocomposites were prepared by a facile precipitation method. The samples were characterized by various measurements. XRD and TEM showed that Er^3+^:CeO_2_ nanoparticles were well-crystallized and loaded on the surface of ATP. The visible light was converted into ultraviolet light by Er^3+^:CeO_2_ as evidenced by upconversion photoluminance (PL) analysis. The mass ratio of Er^3+^:CeO_2_ to ATP on the desulfurization efficiency was investigated. Results showed that the desulfurization rate reached 87% under 4 h visible light irradiation when the mass ratio was 4:10. The mechanism was put forward as follows. Er^3+^:CeO_2_ and ATP formed Z-scheme heterostructure intermediated by oxygen vacancy, leading to the enhanced separation of photogenerated charges and preservation of high oxidation-reduction potential, both of which favored for the generation of radicals to oxidize sulfur species.

## Introduction

Massive use of fuel has given rise to serious environmental problem including acid rain and atmospheric haze, since the combustion of sulfur compounds in fuels leads to the emission of pernicious SO_x_. Therefore, it is imperative to develop new desulfurization technology to satisfy fuel purification (Liu et al., 2017; Zhang et al., 2018). The conventional hydrodesulfurization (HDS) has been extensively used in removing sulfur compound in fuel, which requires high temperature, pressure and expensive hydrogen (Wang et al., 2016; Zeng et al., 2017). Owing to the drawbacks of HDS for removing sulfur compound, many alternative strategies have been developed, including extractive desulfurization (Raj et al., 2017), oxidative desulfurization (Khodadadi Dizaji et al., 2018), biodesulfurization (Agarwal et al., 2016) and adsorption desulfurization (Yang et al., 2018). Among these strategies, oxidative desulfurization is considered as one of the promising strategy for deep desulfurization due to its cheap and efficient features. Particularly, photocatalytic oxidative desulfurization is deemed as a potential candidate. In our previous work, we have prepared attapulgite-CeO_2_/MoS_2_, CeO_2_/attapulgite/g-C_3_N_4_ and BiP_1−x_V_x_O_4_/attapulgite nanocomposites and found that the photocatalytic oxidative desulfurization rate reached as high as more than 90% under the irradiation of visible light (Li et al., 2016, 2017, 2018). As a natural clay material, attapulgite (ATP) has large specific surface area, superior adsorption performance and unique pore structure, which is widely used in catalyst support (Zhang et al., 2016b). Interestingly, the incorporation of Fe_2_O_3_ endows ATP with semiconductor property to some extent. Zhang et al. (Zhang et al., 2013, 2016a) sensitized ATP by taking advantage of eosine Y and CdS to generate hydrogen from water. Li et al. (Ma et al., 2018) prepared CQDs/ATP nanocomposites with visible light response. However, ATP can only be stimulated by ultraviolet light which is accounted for 5% in solar light due to its wide band gap (Balaji et al., 2017).

The upconversion luminescence materials have attracted great attentions since they can absorb and upconvert long wave light with low-energy to short wave light with high-energy (Feng et al., 2013). In the general composition of upconversion luminescence material, the rare earth element has a rich 4f energy level enabling the electrons jump easily from high to low energy level. Among the rare earth family, Er^3+^ has abundant energy levels and higher upconversion luminescence efficiency, making it as a excellent candidate for the conversion from visible to UV light (Pickering et al., 2017). The Er^3+^ can be easily doped into the crystal lattice of CeO_2_ along with the production of oxygen vacancy since the ion radius of Ce^4+^ is very close to Er^3+^ (Wu et al., 2014). Meanwhile, CeO_2_ has high chemical stability and low phonon energy, making it suitable as matrix materials in upconversion. Han et al prepared upconversion Er, Yb-CeO_2_ hollow spheres for improving the efficiency of dye-sensitized solar cells (Han et al., 2017). However, rare report has been put on the heterostructure constructed by rare earth doped upconversion luminescence materials (Bhethanabotla et al., 2016). Moreover, the decline of oxidation-reduction ability in traditional type heterostructure is non-negligible. It is worth noting that Z-type heterostructure causes the annihilation of photo-generated charges with lower reduction and oxidation potential, therefore leading to the preservation of high redox potential for the heterostructure (Šutka et al., 2018). Intriguingly, the self-generated oxygen vacancy has been proposed as mediator in the indirect Z-scheme with the absence of noble metals, such as Au, Ag, etc. For instance, Ding et al. (Ding et al., 2016) prepared BiO_1−x_Br/Bi_2_O_2_CO_3_ in which the oxygen vacancy of BiO_1−x_Br acted as the medium of transmission for electrons and the recombination center of photogenerated electrons and holes.

In this work, the Er doped CeO_2_ upconversion luminescence oxide was immobilized on ATP. The doping fraction of Er^3+^ was adjusted to achieve the strongest emission of ultraviolet light to stimulate ATP. Meanwhile, CeO_2_:Er and ATP formed Z-type heterostructure intermediated by oxygen vacancy, which effectively preserved redox potential so as to improve the photocatalytic desulfurization activity of Er^3+^:CeO_2_/ATP.

## Experimental section

### Materials

ATP powders were obtained from Xuyi, China. Ce(NO_3_)_3_·6H_2_O, Er(NO_3_)_3_·5H_2_O, hexamine (C_2_H_12_N_4_, HMT), octane (C_8_H_18_), dibenzothiophene (C_12_H_8_S) and acetonitrile (C_2_H_3_N) were purchased from Sionpharm Chemical Reagent Co., Ltd. All reagents were analytical grade without further purification.

### Synthesis of Er^3+^:CeO_2_/ATP

Er^3+^:CeO_2_/ATP composites were synthesized via a one-step precipitation method. Typically, adequate amount of Ce(NO_3_)_3_·6H_2_O, Er(NO_3_)_3_·5H_2_O and 1 g ATP were dissolved in 100 mL deionized water and mixed together, followed by adding excess HMT (molar ratio of HMT to Ce(NO_3_)_3_ was 5:1). The molar fraction of Er (Er/Ce+Er) was adjusted from 0.5 to 2.5%, and the mass ratio of Er^3+^:CeO_2_ to ATP was adjusted from 1:10 to 5:10. Then the mixture was heated in a water bath at 80°C for 2 h. After cooled down to room temperature, the precipitate was washed with deionized water for three times. Subsequently, the obtained solid was dried in vacuum at 80°C for 10 h, and finally calcined at 300°C for 2 h.

### Materials characterization

The powder X-ray diffraction (XRD) was performed with a D/max 2500PC diffractometer equipped with a Cu-Kα radiation (λ = 1.5406 Å) at a scanning speed of 6° min^−1^ from 5 to 80°. The morphology was investigated with a JEM-2100 transmission election microscope (TEM) operating at 200 kV. Raman spectra were collected with a Renishaw (UK) spectrometer with an Ar ion laser of 514 nm excitation. The ultraviolet visible (UV–Vis) spectra were acquired using a UV-2500 Shimadzu UV–Vis spectrophotometer. The photoluminescence (PL) spectra were collected with the PerkinElmer LS45 at room temperature. The X-ray photoelectron spectroscopy (XPS) was performed with a PHI 5300 equipped with Kα in the condition of 284.6 eV for C 1s.

### Photocatalytic oxidative desulfurization

The photocatalytic desulfurization performance of Er^3+^:CeO_2_/ATP was carried out by degradating the model gasoline using a photocatalytic reaction apparatus (GHX-2) which was equipped with a 300 W xenon lamp. 0.4 g DBT was dissolved into 500 mL octane to acquire the model gasoline with sulfur compounds of 200 ppm. Then the model gasoline and catalysts were added into the photocatalytic reactor and kept 30 min with magnetic stirring to ensure adsorption equilibrium. Subsequently, simulated solar light using UV-cut off was irradiated during the reaction with the irradiation intensity of 30 W/cm^2^. The samples were collected twice an hour to satisfy the extraction process. The rest of sulfur content was measured by a sulfur determination device (THA2000S), and the desulfurization rate D was calculated based on the following formula:

$$\begin{array}{l} \text{D }=\;\left(1-\frac{C}{C_{0}}\right)\;\times \;100\% \end{array}$$

where C_0_ is the initial sulfur content of the model gasoline and C is the final sulfur content.

## Results and discussions

### XRD analysis

**Figure 1:**
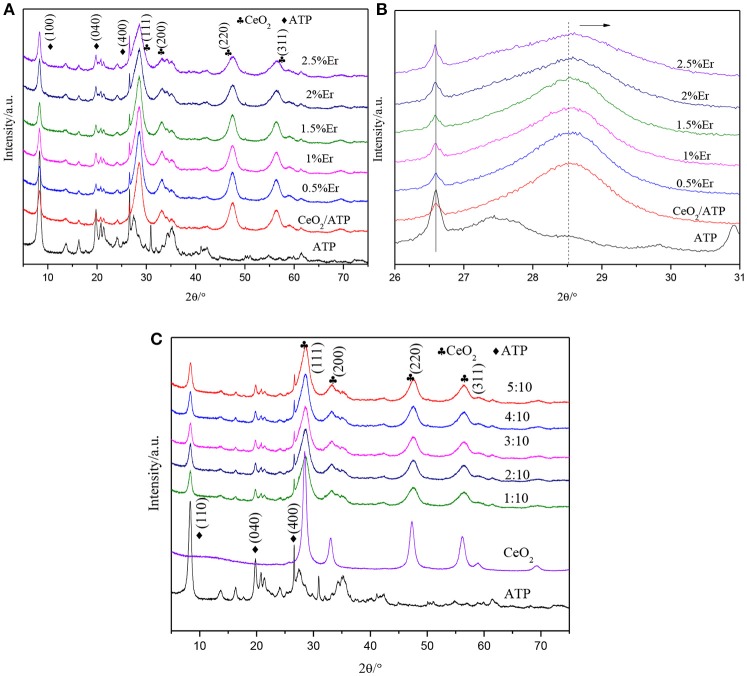
XRD patterns of ATP, CeO_2_/ATP and Er^3+^:CeO_2_/ATP with various molar fractions of Er doping **(A)**, the enlarged diffraction peak from 26 to 31° **(B)**, and different mass ratio of Er^3+^:CeO_2_ to ATP **(C)**.

Figure 1 shows the XRD patterns of ATP, CeO_2_/ATP and Er^3+^:CeO_2_/ATP. In Figure 1A the characteristic peaks at 8.21, 19.75, and 26.54°Correspond to the (110), (040), and (400) plane of pure ATP (Zhao et al., 2016). The characteristics at 28.55, 33.07, 47.48, and 56.34°Correspond to the (111), (200), (220), and (311) plane of CeO_2_ (JCPDS 43-1002). However, there is no characteristic peak of Er in Er^3+^:CeO_2_/ATP composites, which may be due to the small doping amount of Er. In Figure 1B, the characteristic peak at 28.55° shows slight shift to higher Bragg angle, implying that the Er^3+^ dopant results in the lattice contraction due to the fact that Er^3+^ (0.88 Å) replaced the Ce^4+^ (0.92 Å), subsequently leading to the lattice distortion and formation of oxygen vacancies (Li et al., 2012). Figure 1C shows the XRD patterns of different mass ratio of Er^3+^:CeO_2_ to ATP. With the increase of mass ratio, the characteristic peak intensity of ATP is gradually weakened whereas the intensity of CeO_2_ is strengthened without any change of peak position, suggesting the immobilization of Er^3+^:CeO_2_ on ATP.

### Raman analysis

**Figure 2:**
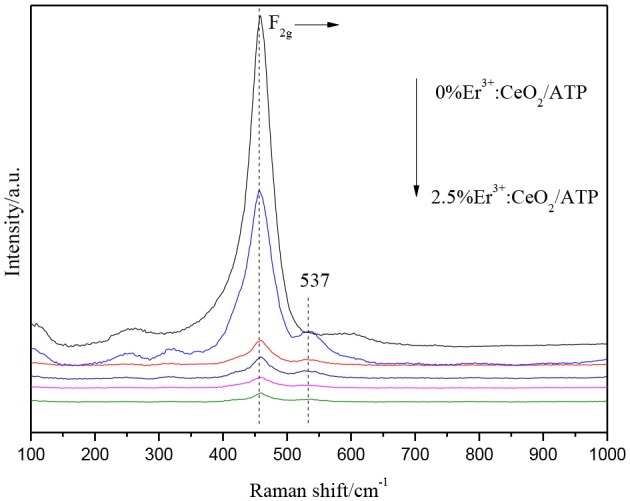
Raman patterns of CeO_2_/ATP and Er^3+^:CeO_2_/ATP.

Figure 2 shows the Raman patterns of CeO_2_/ATP and Er^3+^:CeO_2_/ATP. The peaks appearing in 454–459 cm^−1^ represent the F_2g_ vibration mode for cubic CeO_2_ (Kumar and Kumar, 2017). Meanwhile, the peak intensity of F_2g_ is gradually decreased and the peak position is shifted to high wavenumber with the increase of Er, implying that Er is doped into CeO_2_ lattice. The peaks located at 537 cm^−1^ can be attributed to the formation of oxygen vacancies (Mamontov et al., 2016), due to the fact that Er^3+^ replaces Ce^4+^ resulting in the transformation from Ce^4+^ to Ce^3+^, subsequently oxygen vacancies are produced which is consistent with the results of XRD.

### Optical analysis

**Figure 3:**
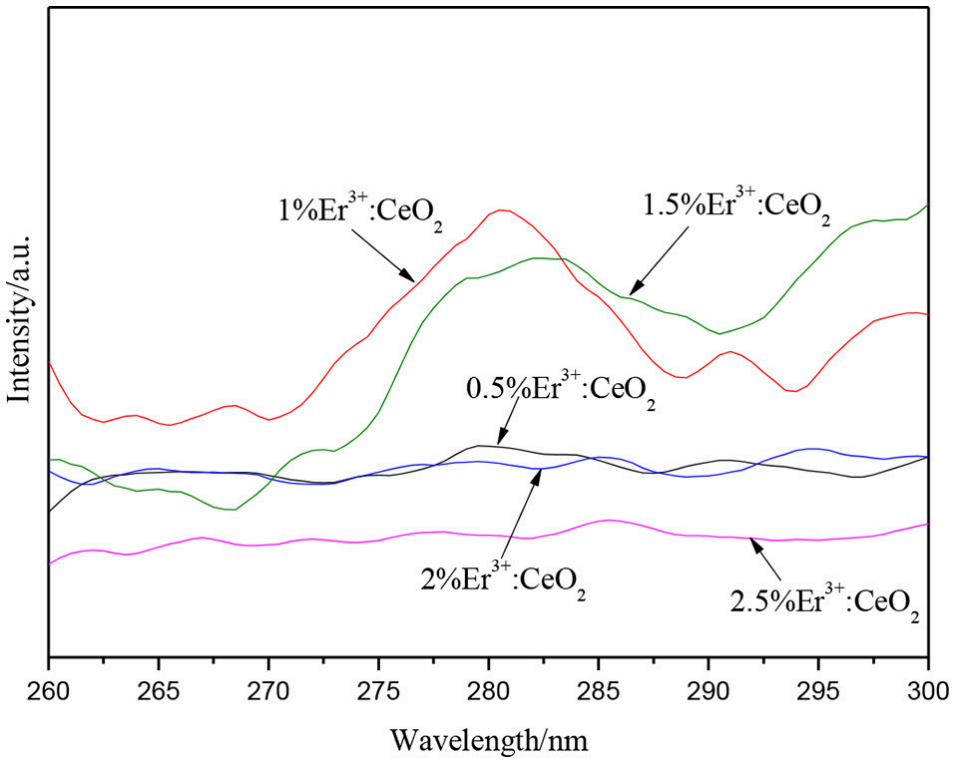
Upconversion PL patterns of different Er doping fraction.

Figure 3 demonstrates the upconversion PL spectra of Er^3+^:CeO_2_ with different Er molar fraction. The upconversion luminescence was tested by the visible light of 488 nm as excitation light, while ultraviolet light was emitted near 281 nm, corresponding to the energy transfer from the 2D_5/2_ excited state to the 4I_15/2_ ground state. The upconversion luminescence intensity of Er^3+^:CeO_2_ is gradually increased below 1%, which reach strongest when the doping fraction is 1%. Afterwards, the upconversion luminescence intensity is obviously decreased when doping fraction is more than 1% due to the fact that the excessive doping of Er^3+^ shortens the distance and enhances the interaction among Er^3+^ ions, leading to the concentration quenching of the upconversion luminescence (Ramasamy et al., 2013). Therefore, the upconversion luminescence intensity is gradually decreased, and the doping fraction is optimized to be 1%.

### TEM analysis

**Figure 4:**
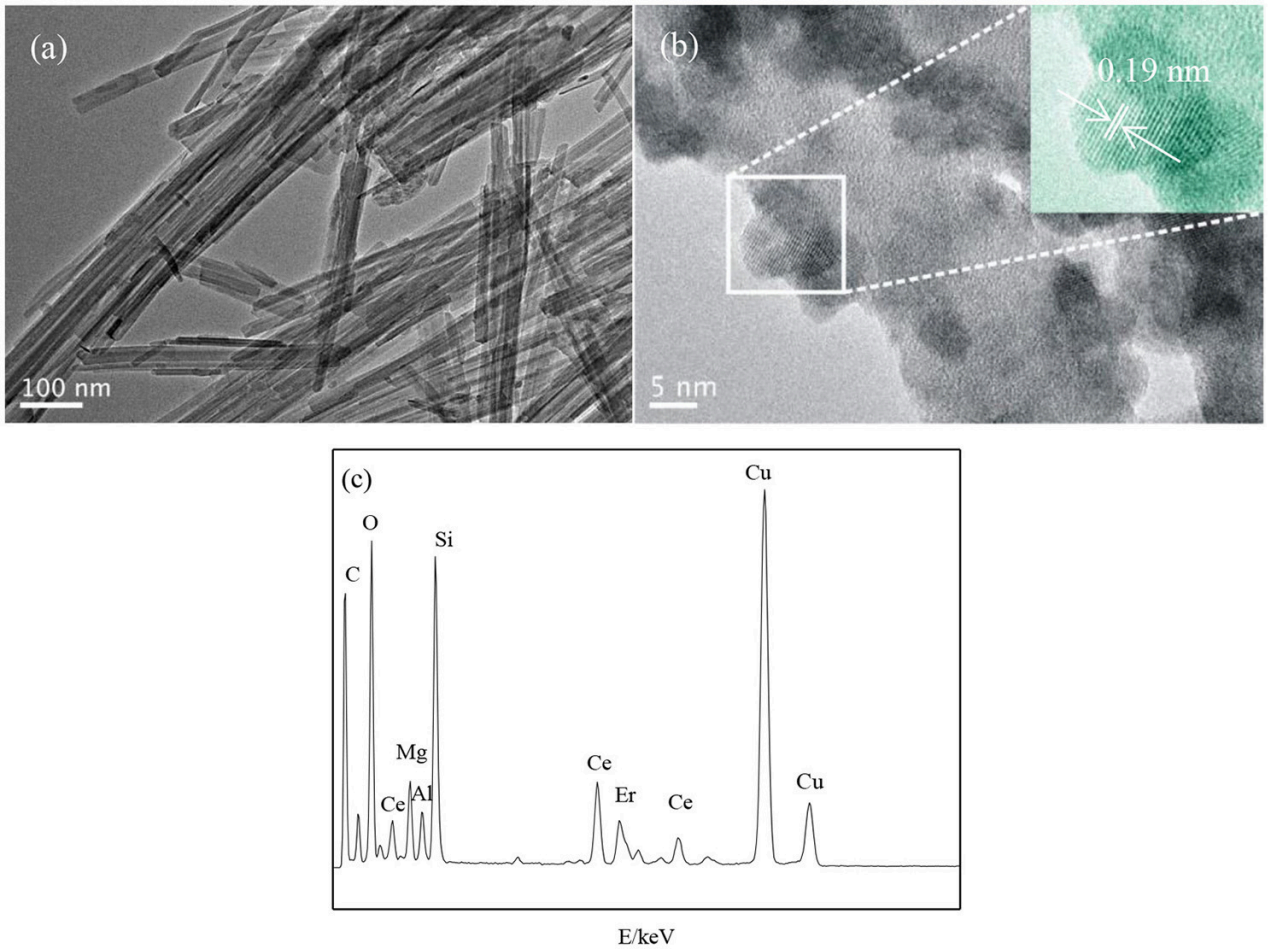
TEM images of ATP **(a)**, Er^3+^:CeO_2_/ATP **(b)**, and EDS of Er^3+^:CeO_2_/ATP **(c)**.

Figure 4 shows the TEM results of ATP and Er^3+^:CeO_2_/ATP. Figure 4a shows the pure ATP with a rod like structure having average diameter of 20–30 nm. Figure 4b shows the HRTEM image of 1% Er^3+^:CeO_2_/ATP (4:10), and apparent nanoparticles are loaded on the surface of ATP. The inset lattice distance of CeO_2_ is 0.19 nm corresponding to the (220) plane of CeO_2_. Energy-dispersive spectroscopy (EDS) in Figure 4c displays Ce, Er, Si, Fe, Mg, and Al elements, in which Si, Fe, Mg and Al are derived from ATP.

### UV–Vis analysis

**Figure 5:**
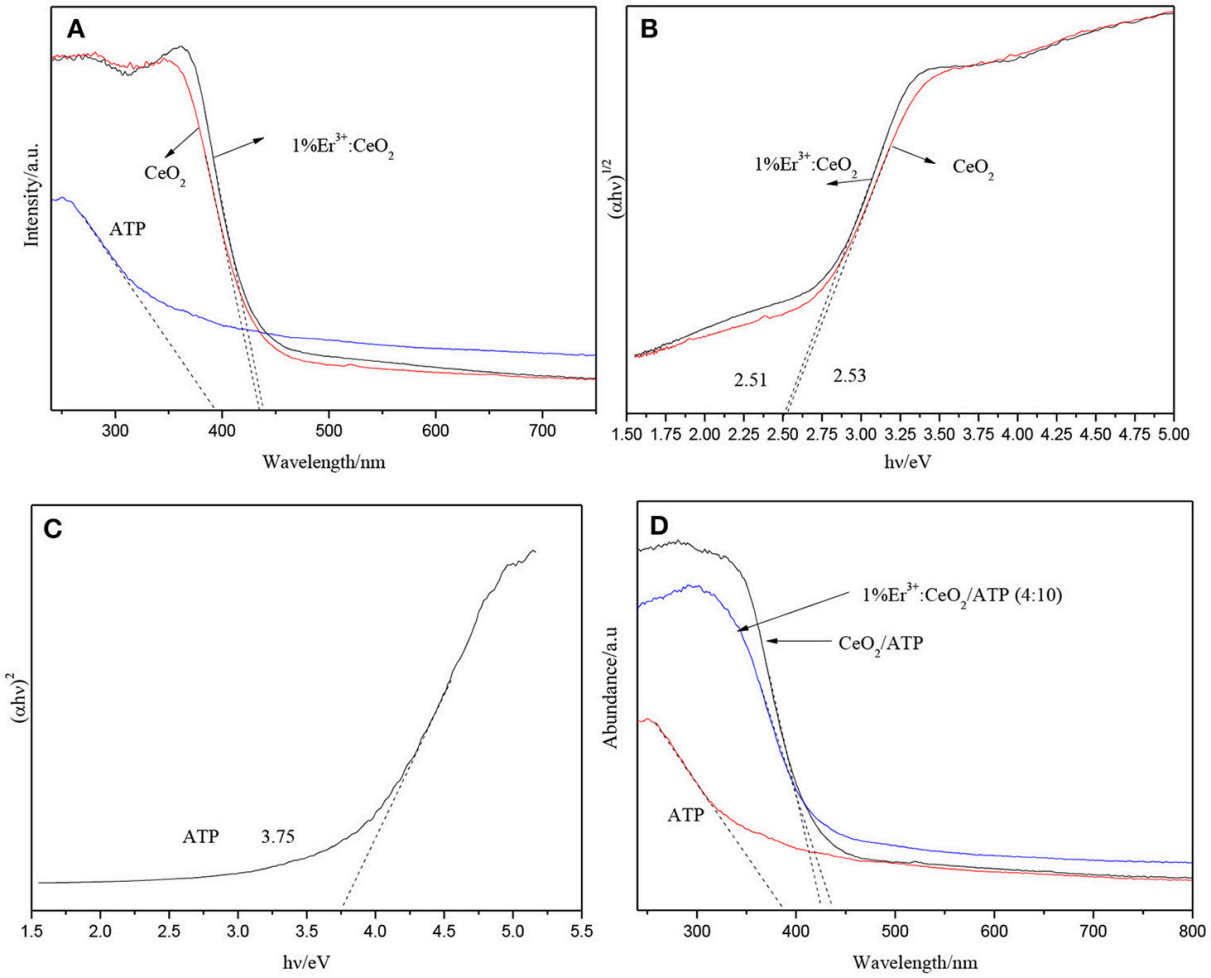
UV–Vis patterns of ATP, CeO_2_, and 1%Er^3+^:CeO_2_ **(A)**, the plots of transformed Kubelka-Munk function vs. light energy of CeO_2_, and 1%Er^3+^:CeO_2_ **(B)**, the plots of transformed Kubelka-Munk function vs. light energy of ATP **(C)**, and the UV–Vis patterns of ATP, CeO_2_, and 1%Er^3+^:CeO_2_ (4:10) **(D)**.

Figure 5A shows the UV–Vis image of CeO_2_, ATP, and Er doping fraction for 1%. CeO_2_ has a certain response to visible light, while the absorption edge of CeO_2_ appears slightly red shift after doping of Er, which due to the fact that Er doping may change the band gap of the CeO_2_. Figure 5B shows the plots of transformed Kubelka-Munk function vs. light energy of CeO_2_ and 1% Er^3+^:CeO_2_. The band gap of CeO_2_ and 1% Er^3+^:CeO_2_ is estimated to be 2.53 and 2.51 eV, respectively. As shown in Figure 5C, the band gap of ATP is estimated to be 3.75 eV, indicating ATP has ultraviolet response ability. It is reported that a semiconductor absorbs energy which is equal or larger than its band gap to produce photogenerated e^−^ and h^+^ (Zhuo et al., 2012). According to the results in Figure 3, the upconversion wavelength of 1% Er^3+^:CeO_2_ locates at 281 nm corresponding to the band gap of 4.41 eV larger than the band gap of ATP with 3.75 eV. Therefore, ATP can be excited by the upconversion emission of 1% Er^3+^:CeO_2_. Figure 5D shows the UV–Vis pattern of ATP, CeO_2_/ATP and 1%Er^3+^:CeO_2_/ATP. Compared with ATP, the absorption edge of CeO_2_/ATP and 1% Er^3+^:CeO_2_/ATP shows obviously red shift, which may favor for the photocatalytic performance.

### PL analysis

**Figure 6:**
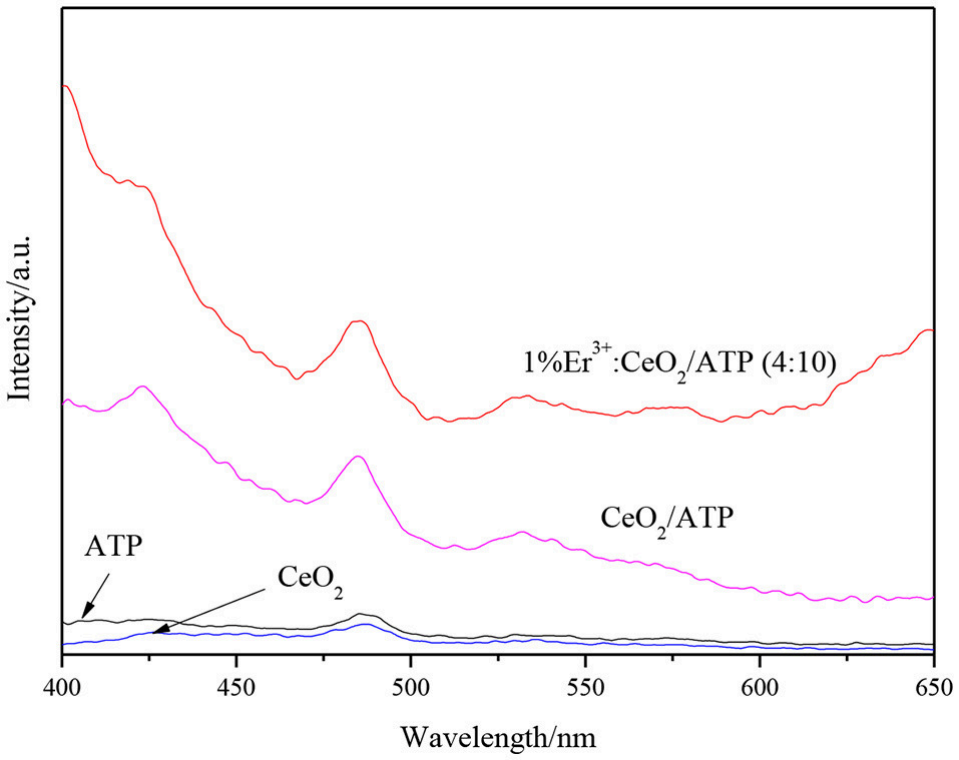
PL patterns of ATP, CeO_2_, CeO_2_/ATP, and 1%Er^3+^:CeO_2_/ATP (4:10).

In order to investigate the recombination effect of photogenerated electrons and holes, PL analysis is performed under excitation of 300 nm. Figure 6 shows the PL patterns of ATP, CeO_2_, CeO_2_/ATP and 1% Er^3+^:CeO_2_/ATP (4:10). The emission peak of ATP is displayed, indicating that the photogenerated electrons and holes were recombined due to the fact that few Fe_2_O_3_ is stimulated to produce the photogenerated electrons and holes. Compared with pure ATP and CeO_2_, the intensity of the emission peak of CeO_2_/ATP and 1%Er^3+^:CeO_2_/ATP (4:10) is significantly increased. In general, the intensity of the emission peak of PL is inversely proportional to the separation efficiency of the photogenerated charges. Obviously, the phenomenon described in the Figure 6 violates the traditional law, which may be due to the unique Z-type heterostructure formed by Er^3+^:CeO_2_ and ATP.

### XPS analysis

**Figure 7:**
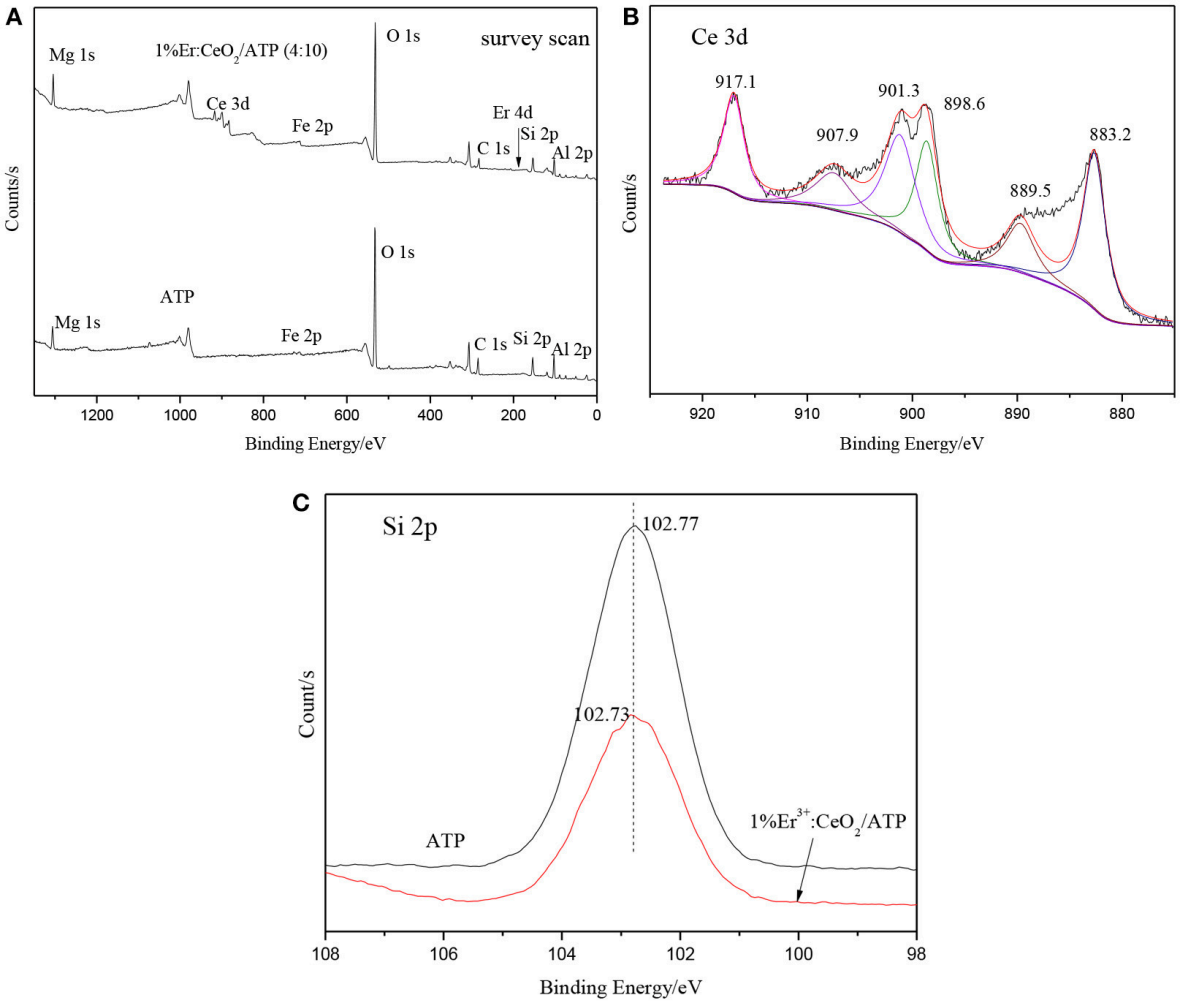
XPS patterns of ATP, and 1%Er^3+^:CeO_2_/ATP (4:10), **(A)** Survey scan, **(B)** Ce 3d, and **(C)** Si 2p.

Figure 7 shows the XPS patterns of ATP and 1%Er^3+^:CeO_2_/ATP (4:10). Figure 7A is the survey scan indicating the existence of Mg, Fe, Si, Al, Ce, Er, and C elements where Mg, Fe, Si, and Al are originated from ATP, Ce and Er are originated from Er^3+^:CeO_2_. As shown in Figure 7B, the characteristic peaks of 883.2, 898.6, and 907.9 eV are ascribed to Ce^3+^ while the characteristic peaks of 889.5, 901.3, and 917.1 eV are ascribed to Ce^4+^, which is close to our previous result with respect to Ce 3d in CeO_2_/ATP, (Li et al., 2017) indicating that the surface of CeO_2_ contains oxygen vacancy. (Peng et al., 2017) The characteristic peak position of Si 2p in 1% Er^3+^:CeO_2_/ATP (4:10) is lower than that of ATP shown in Figure 7C, due to the fact that the Si-O-Si bond may be replaced by Si-O-Ce bond since the electronegativity of Ce is less than that of Si.

### Photocatalytic oxidation desulfurization

**Figure 8:**
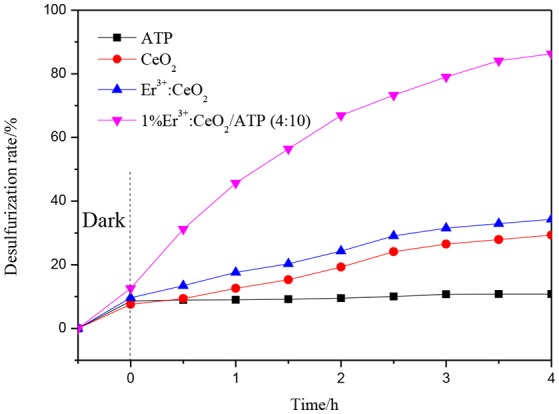
Photocatalytic oxidation desulfurization efficiency with various catalysts.

The photocatalytic oxidation desulfurization is performed using various catalysts. As shown in Figure 8, the desulfurization rate is 10.8, 29.3, and 34.3% corresponding to ATP, CeO_2_ and 1%Er^3+^:CeO_2_ samples. Meanwhile, the photocatalytic oxidation desulfurization rate of 1%Er^3+^:CeO_2_/ATP (4:10) reaches 87%. The 1%Er^3+^:CeO_2_ and ATP may have synergy which accelerates the migration of photogenerated electrons and holes. Furthermore, a unique Z-type structure may form perseving the high redox potential, thus leads to the improved desulfurization performance.

**Figure 9:**
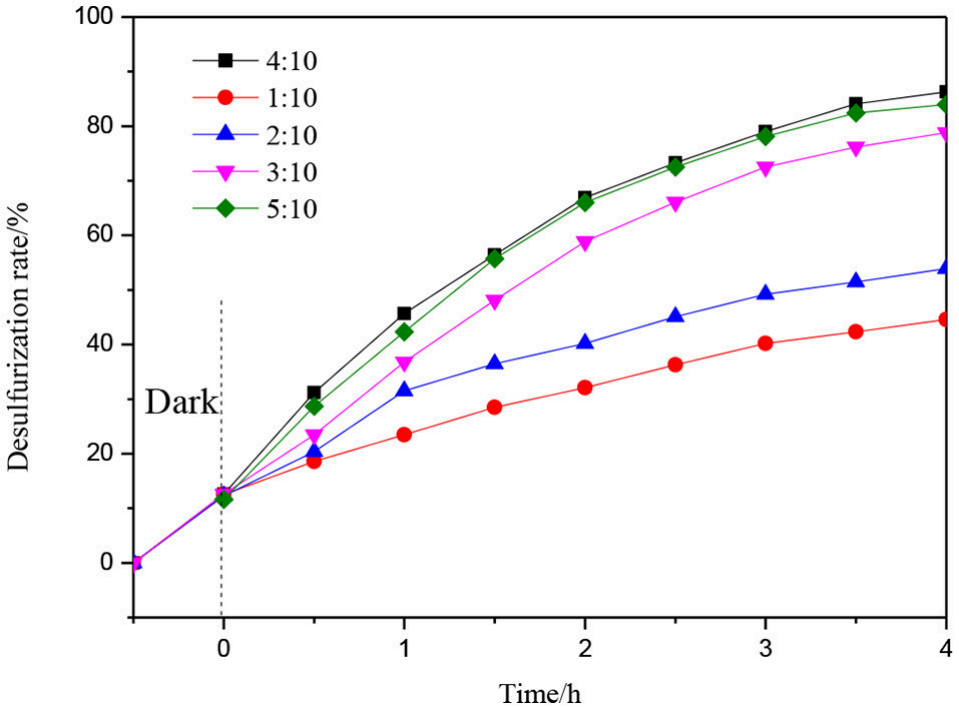
Photocatalytic desulfurization efficiency with different mass ratio of 1%Er^3+^:CeO_2_, and ATP.

Figure 9 shows the photocatalytic desulfurization performance with different mass ratios of 1%Er^3+^:CeO_2_ and ATP. The desulfurization rate is enhanced gradually with the increase of the mass ratio. When the mass ratio is 4:10, the photocatalytic desulfurization rate of 1%Er^3+^:CeO_2_/ATP reached 87% within 4 h, which may be due to the fact that with the content of 1%Er^3+^:CeO_2_ increased, the concentration of oxygen vacancy is increased favoring for the transmission of charges. When the mass ratio is more than 4:10, the photocatalytic desulfurization rate is decreased, since the excess Er^3+^:CeO_2_ nanoparticles may lead to aggregation restraining the exposure of surface active sites and destroying the balance of recombination of charges.

### Photocatalytic desulfurization mechanism

**Figure 10:**
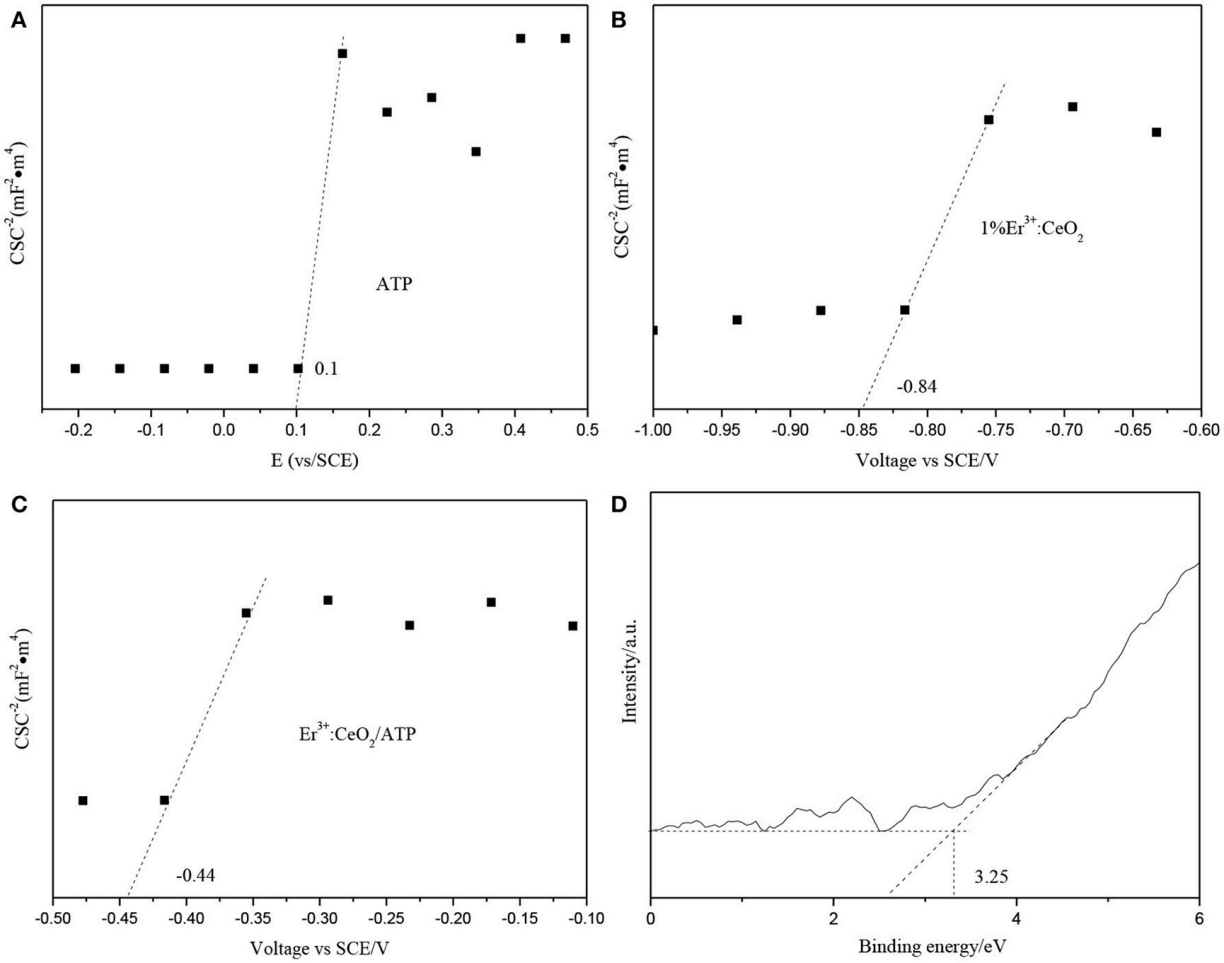
Mott-schottky patterns of ATP **(A)**, 1%Er^3+^:CeO_2_ **(B)**, 1%Er^3+^:CeO_2_/ATP (4:10) **(C)**, and VB-XPS pattern of ATP **(D)**.

The position of conduction band (CB), valence band (VB) and Fermi energy levels (E_f_) of 1%Er^3+^:CeO_2_ and ATP were calculated, respectively. Figures 10A,B show the Mott-schottky patterns of ATP and 1%Er^3+^:CeO_2_, where the flat band tential (identical E_f_) of ATP and 1%Er^3+^:CeO_2_ is determined to be 0.1 and −0.84 eV, respectively. Figure 10C shows the Mott-schottky pattern of 1%Er^3+^:CeO_2_/ATP (4:10) after contact, and the equilibrium E_f_ level is shifted to −0.44 eV located between that of Er^3+^:CeO_2_ and ATP. Figure 10D shows the VB-XPS pattern of ATP, in which the VB value of ATP is determined to be 3.25 eV. In addition, the empirical formula of VB and CB is as follows (Obregón et al., 2016):

$$\begin{array}{l} {\text{E}}_{\text{VB}}\;=\text{ X}-E^{e}+\frac{1}{2}E_{g}\\ {\text{E}}_{\text{CB}}\;=\;E_{VB}-E_{g} \end{array}$$

where E_VB_ and E_CB_ represent the VB and CB edge potential, respectively, X represents the electronegativity, E^e^ (about 4.5 eV) is the energy of free electrons on the hydrogen, and E_g_ is the band gap of the semiconductor. According to the above formula, the VB value for 1%Er^3+^:CeO_2_ is calculated to be −0.42 eV. Since the E_g_ of ATP and 1%Er^3+^:CeO_2_ is 3.75 and 2.51 eV by UV–Vis analysis in Figure 5, the corresponding CB for ATP and 1%Er^3+^:CeO_2_ is −0.5 and −2.93 eV, respectively.

**Figure 11:**
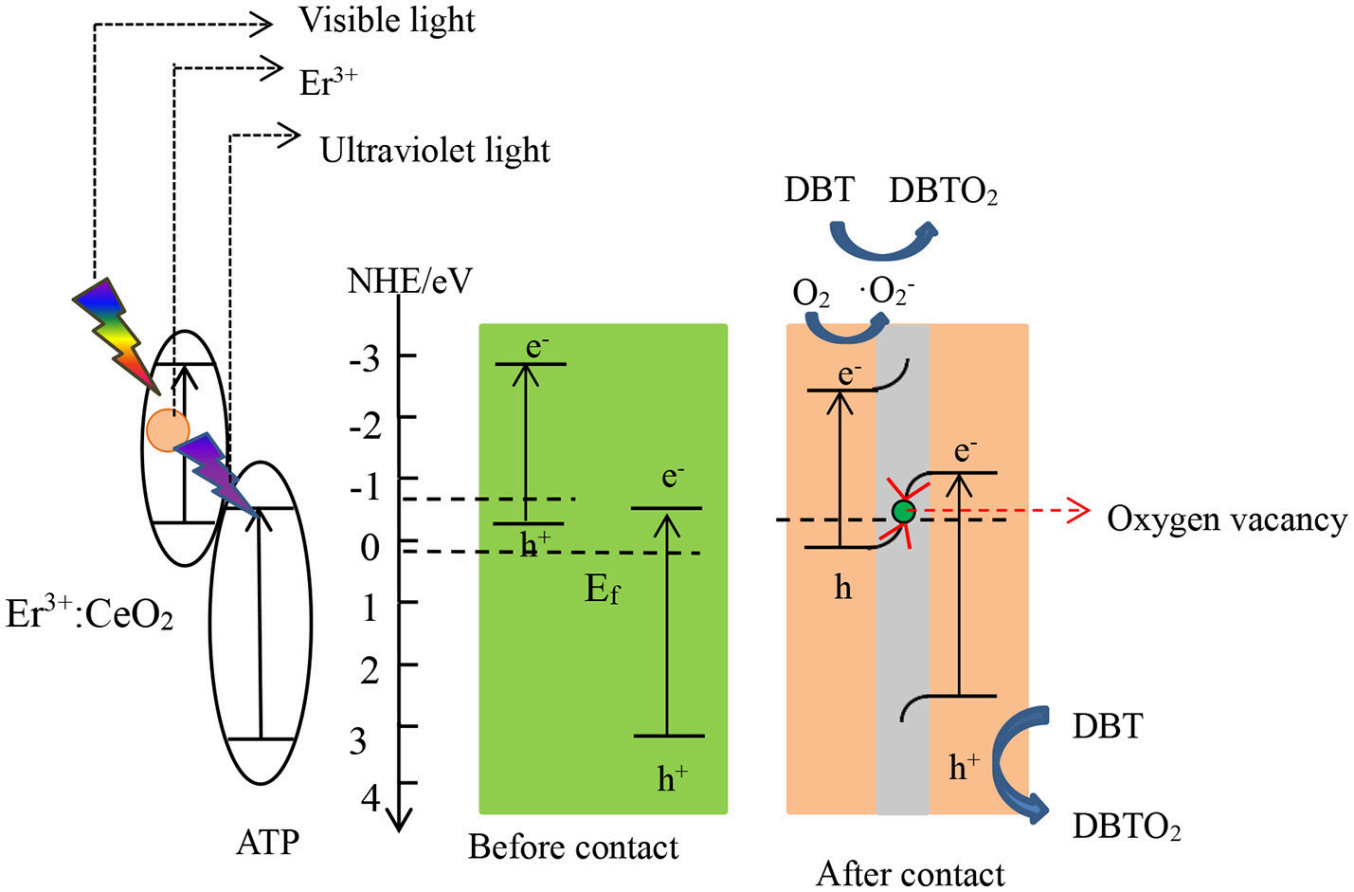
Photocatalytic desulfurization mechanism of Er^3+^:CeO_2_/ATP.

According to the above analysis, the photocatalytic desulfurization mechanism of 1%Er^3+^:CeO_2_/ATP (4:10) is proposed as shown in Figure 11. After contact, free electrons may flow from 1%Er^3+^:CeO_2_ to ATP due to the disparity of E_f_ until they reach equilibrium, which forms an internal electric field, leading to the consumption and accumulation of free electrons (Huang et al., 2017). Simultaneously, the energy band edge of Er^3+^:CeO_2_ is bended upward whereas the energy band edge of ATP is bended downward. Under visible light irradiation, the 1%Er^3+^:CeO_2_ is stimulated to produce photogenerated e^−^ and h^+^ while Er^3+^ converts visible light to ultraviolet light. Subsequently, ATP is stimulated by the upconverted ultraviolet light to produce photogenerated e^−^ and h^+^. Then the downward band bending of ATP allows e^−^ flow to the oxygen vacancy in CeO_2_ while the upward band bending of 1%Er^3+^:CeO_2_ allows h^+^ flow to the oxygen vacancy which acted as the recombination center for the e^−^ and h^+^ (Ding et al., 2016). Finally, the photogenerated e^−^ in the CB of 1%Er^3+^:CeO_2_ is preserved and reacts with O_2_ to produce ·${\text{O}}_{2}^{-}$. Then, ·${\text{O}}_{2}^{-}$ and h^+^ in the VB of ATP synergistically oxidize DBT to DBTO_2_ (Li et al., 2018). According to the above statement, the reaction equations are put forward as follows:

(1) $$\begin{array}{r} 1\%\text{E}{\text{r}}^{3+}:\text{Ce}O_{2}/\text{ATP }+\text{ hv }\to \;e^{-}\;+\;h^{+} \end{array}$$

(2) $$\begin{array}{r} {\text{e}}^{-}\;+\;O_{2}\;\to \;{\cdot O}_{2}^{-} \end{array}$$

(3) $$\begin{array}{r} {\cdot {\text{O}}_{2}}^{-}\;+\text{ DBT }\to \;DBTO_{2} \end{array}$$

(4) $$\begin{array}{r} {\text{h}}^{+}\;+\text{ DBT }\to \;DBTO_{2} \end{array}$$

## Conclusion

In this work, Er^3+^:CeO_2_/ATP nanocomposites have been successfully prepared via a one-step precipitation method. Doping of Er not only alters the band gap of CeO_2_, but also converts visible light to ultraviolet light and reach the strongest when the doping fraction of Er is 1%. Er^3+^:CeO_2_ and ATP form Z-type heterostructure intermediated by oxygen vacancy, which promotes the transfer of photogenerated electrons and holes and preserves the charges with higher oxidation-reduction ability. The photocatalytic desulfurization rate reaches the highest 87% when the mass radio of Er^3+^:CeO_2_ and ATP is 4:10. This novel photocatalyst integrated by natural clay and rare earth upconversion may pave a new way for design of eco-friendly materials and beyond.

## Author contributions

FW drafted the manuscript, XL made analysis and revision, HZ synthesized the samples and conducted desulfurization experiment, SZ characterized the samples, CY provided the idea and proposed the mechanism.

### Conflict of interest statement

The authors declare that the research was conducted in the absence of any commercial or financial relationships that could be construed as a potential conflict of interest.
